# For the Medical and Physical Journal

**Published:** 1801-02

**Authors:** 


For the MEDICAL and PHYSICAL JOURNAL.

THE introduction of the Vaccine Inoculation in the populous towns of Plymouth and Plymouth Dock, at a very early period after the commencement of the practice in this country, and the continuance of it on an extenfive fcale to the prefent time, have afforded fufficient opportunities to a numerous body of practitioners to confirm by their own obfervations, and by the teftimony of their immediate friends, the ample and refpectable evidence which has been adduced in favour of this eminently ufeful difcovery. The general fuccefs of the new Inolation has not, however, prevented the circulation of reports of unfavourable accidents, and of occurrences inconfiftent with its character of abfolute fafety in itfelf, and of affording permanent fecurity againft the future invafion of Small-pox. As the prevalence of fuch reports muft have a tendency to excite doubts of the fafety and efficacy of the new Inoculation in the minds of thofe who have not the opportunity of tracing them to their origin, and who are not acquainted with the ftrength and folidity of the mafs of evidence on which the fupporters of the new practice ground their conviction of its utility, we have thought, that it might not be unattended with advantage to the public, at leaft to that fmall part of it to which our names and characters are known, to add to the numerous atteftations already given, a Declaration, as well of our conviction of the efficacy and fecurity of the practice of inoculating the Cow-pox in general, as of our full perfuafion, refulting from an inquiry into the circumftances of the cafes adduced, that no inftance has occurred in this neighbourhood, which is entitled in any degree to call in queftion the reafonablenefs of this conviction, the reports which have arifen being evidently founded in error and mifreprefentation. The effects of mifreprefentation can be obviated only as they arife; but it may not be amifs to obferve here more particularly, that the Small-pox has lately prevailed prevailed in this neighbourhood to a confiderable degree, and that, in all the inftances in which this difeafe is faid to have occurred after inoculation for the Cow-pox, the infection of the former was evidently received before the inoculation for the latter, or the operation had failed of producing thofe fymptoms of conftitutional affection, on which alone fecurity depends. The fources of error will, we truft, be in future avoided, by due attention to thofe cautions in the practice, which have been fo fully pointed out by Dr. Jenner and others.

*Plymouth and Plymouth Dock, Jan.* 7, 1801.

R. B. Remmett, M. D.
William Woollcombe, M. D.
T. Trotter, M. D.
R. Sheppard, M. D.
James Gafking, M. D.
Robert Fortefcue,
James Courtenay,
George Cooban,
Richard Dunning,
D. Little,
Vaughan May,
Robert Sargent,
Andrew H. Sargent,
Thomas Stewart,
John Smith,
D. W. Spry,
J. Lower,
John Penkivil,
J. Rivers Lancafter,
Samuel Fuge,
Thomas Thynne Folds,
Stephen Hammick,
Stephen Hammick, jun.
Nathaniel Seecombe,
Thomas Richardfon,
Richard Jones,
J. Beard Samhill,
R. H. Armftrong,
W. Bragg,
Peter Rowe,
John Bone.

